# Analysis of image data from the EuroNet PHL-C2 trial indicates a potential reduction in injected F-18 FDG activities in children: a proposal to update the EANM Paediatric Dosage Card

**DOI:** 10.1007/s00259-023-06396-w

**Published:** 2023-09-20

**Authors:** Johannes Tran-Gia, Uta Eberlein, Michael Lassmann, Christine Mauz-Körholz, Dieter Körholz, Pietro Zuccetta, Zvi Bar-Sever, Ute Rosner, Thomas Walter Georgi, Osama Sabri, Regine Kluge, Arnoldo Piccardo, Lars Kurch

**Affiliations:** 1 Department of Nuclear Medicine, University Hospital Würzburg, Würzburg, Germany; 2 Department of Paediatric Oncology, Justus-Liebig-University Giessen, Giessen, Germany; 3 Nuclear Medicine Unit, Department of Medicine – DIMED, University Hospital of Padua, Padua, Italy; 4 Schneider Children’s Medical Center, Petach Tikva, Israel; 5 Department of Nuclear Medicine, University Hospital Leipzig, Leipzig, Germany; 6 Department of Nuclear Medicine, E.O. “Ospedali Galliera”, Genoa, Italy

**Keywords:** EANM Paediatric Dosage Card, [^18^F]FDG-PET, [^18^F]FDG dosage, Activity reduction, Radiation protection, EuroNet-PHL-C2 trial, New activity recommendation for [^18^F]FDG-PET

## Abstract

**Background:**

The aim of this work is to provide the currently missing evidence that may allow an update of the Paediatric Dosage Card provided by the European Association of Nuclear Medicine (EANM) for conventional PET/CT systems.

**Methods:**

In a total of 2082 consecutive [^18^F]FDG-PET scans performed within the EuroNet-PHL-C2 trial, the administered [^18^F]FDG activity was compared to the activity recommended by the EANM Paediatric Dosage Card. None of these scans had been rejected beforehand by the reference nuclear medicine panel of the trial because of poor image quality. For detailed quality assessment, a subset of 91 [^18^F]FDG-PET scans, all performed in different patients at staging, was selected according to pre-defined criteria, which (a) included only patients who had received substantially lower activities than those recommended by the EANM Paediatric Dosage Card, and (b) included as wide a range of different PET systems and imaging parameters as possible to ensure that the conclusions drawn in this work are as generally valid as possible. The image quality of the subset was evaluated visually by two independent readers using a quality scoring system as well as analytically based on a volume-of-interest analysis in 244 lesions and the healthy liver. Finally, recommendations for an update of the EANM Paediatric Dosage Card were derived based on the available data.

**Results:**

The activity recommended by the EANM Paediatric Dosage Card was undercut by a median of 99.4 MBq in 1960 [^18^F]FDG-PET scans and exceeded by a median of 15.1 MBq in 119 scans. In the subset analysis (*n* = 91), all image data were visually classified as clinically useful. In addition, only a very weak correlation (*r* = 0.06) between activity reduction and tumour-to-background ratio was found. Due to the intended heterogeneity of the dataset, the noise could not be analysed statistically sound as the high range of different imaging variables resulted in very small subsets. Finally, a suggestion for an update of the EANM Paediatric Dosage Card was developed, based on the analysis presented, resulting in a mean activity reduction by 39%.

**Conclusion:**

The results of this work allow for a conservative update of the EANM Paediatric Dosage Card for [^18^F]FDG-PET/CT scans performed with conventional PET/CT systems.

**Graphical abstract:**

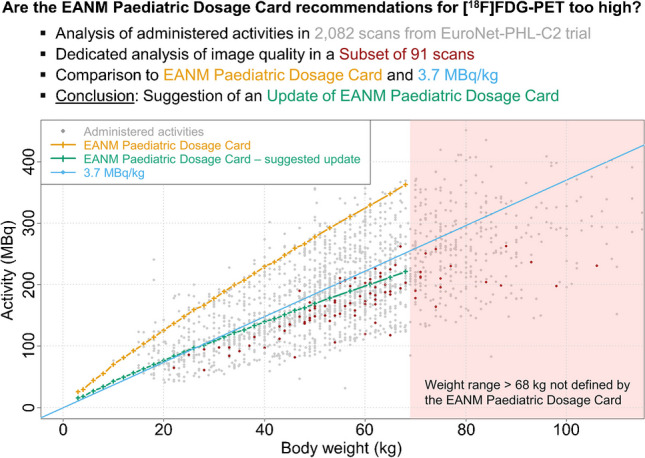

**Supplementary Information:**

The online version contains supplementary material available at 10.1007/s00259-023-06396-w.

## Introduction

The choice of radiopharmaceutical activities in molecular imaging of paediatric patients always involves a trade-off between adequate image quality and radiation exposure. Therefore, the EANM Paediatric Dosage Card and the North American consensus guidelines for radiopharmaceutical activities in paediatrics were developed in 2007 and 2011, respectively, providing recommendations on activities for different radiotracers [1–3]. Despite several updates [4–6], these recommendations have repeatedly been criticised by nuclear medicine specialists, as, in their view, the [^18^F]FDG activities were set too high [7]. Because no evidence could be presented to support their view that lower [^18^F]FDG activities result in good image quality, the recommendations were left unchanged. Based on the original [^18^F]FDG-PET data, collected within a large international multicentre trial using [^18^F]FDG-PET for staging and restaging of Hodgkin lymphoma patients [7], we aimed at providing the missing evidence and at updating the EANM Paediatric Dosage Card for conventional PET/CT systems.

## Methods

The present analysis is based on 2082 consecutive [^18^F]FDG-PET scans from the EuroNet-PHL-C2 trial (EudraCT number: 2012–004053-88; Clinical Trials.gov Identifier: NCT02684708), in which more than 2800 children, adolescents and young adults with Hodgkin’s lymphoma were treated between 2015 and 2021. After [^18^F]FDG-PET imaging at one of the more than 200 paediatric oncology centres located in Europe, Israel, Australia and New Zealand, reference reading was centrally performed at Leipzig University Hospital by experienced nuclear medicine physicians [8]. As the study protocol did not define mandatory criteria on how to perform the [^18^F]FDG-PET scans, the image data closely reflect the clinical reality in various PET centres. More information on the EuroNet-PHL-C2 trial can be found in [9] and in the supplement.

To select a subset of data suitable for the planned analysis, the injected activities recommended by the EANM Paediatric Dosage Card were determined for all 2082 scans based on administered [^18^F]FDG activity and patient weight. Then, a subset of 91 scans was selected, which met the following criteria:

1. At least 70 MBq activity reduction compared to the EANM Paediatric Dosage Card recommendation.
2. Image acquisition for initial staging as lymphoma lesions respond rapidly to chemotherapy. Thus, in [^18^F]FDG-PET images for response assessment minimal uptake due to favourable response to chemotherapy or due to reduced administered [^18^F]FDG activity cannot be distinguished.
3. Broadest possible spectrum of different PET/CT systems. However, if criteria 1 and 2 resulted in more than five scans performed on equivalent systems, all further scans on those systems were skipped.

For further analysis, the percentage activity reduction ∆*A*% for this subgroup was calculated as:

$$\Delta A\%=\frac{{A}_{\text{EANM Paediatric Dosage Card}}-{A}_{\text{Administered}}}{{A}_{\text{EANM Paediatric Dosage Card}}}\cdot 100$$

In addition, mean and standard deviation of the standardised uptake value (SUV) were determined in sphere volumes of interest (VOIs) in “tumour” (1 ml volume around up to 3 lesions) and “background” (30 ml volume in healthy liver tissue). These were used to calculate the tumour-to-background ratio (TBR) as the quotient of mean SUV in the lesion VOI and the liver background VOI. As quantitative measure for the overall noise in the image, the coefficient of variation (CoV) was calculated as the quotient of standard deviation and mean SUV in the liver background VOI.

Lastly, a dedicated image quality assessment was performed by two experienced nuclear medicine physicians based on a visual quality score (QSV). It was defined by the homogeneity of the liver uptake and the visual impression of tumour-to-background in neck and mediastinum and has a value range between 0 (not suitable for clinical reporting) and 9 (excellent image quality).

More information on the data included in the analysis and the determination of TBR, CoV and QSV are provided in the supplement (including Supplemental Table 1). All post-processing and statistics were performed in RStudio 2022.07.2.

## Results

### Characteristics of all 2082 consecutive [^18^F]FDG-PET scans

Of the 2082 [^18^F]FDG-PET scans, 996 had been performed for initial staging (47.8%), 893 were early response (42.9%) and 193 were late response scans (9.3%). The underlying patient population had a wide range of weights (median 55 kg, range 10–140 kg). The median patient age was 15 years (range 2–24 years), with 15.3% younger or equal to 10 years, 82.4% between 11 and 18 years, and 2.3% between 18 and 24 years. In 1960 (94.1%) of the 2082 PET scans, the administered activity was below the recommended activity (median 99.4 MBq, range 0.4–279.8 MBq). In 119 (5.7%) PET scans, the recommended activity was exceeded by a median of 15.1 MBq (range 0.5–126.0 MBq). In the remaining three PET scans, the recommended activity had been administered. Detailed information on the patient characteristics including histograms of patient weight, patient age and administered/recommended activities can be found in the supplement (including Supplemental Fig. 1).

### Analysis of the selected subset of 91 [^18^F]FDG-PET scans

#### Characteristics of the subset

**Fig. 1:**
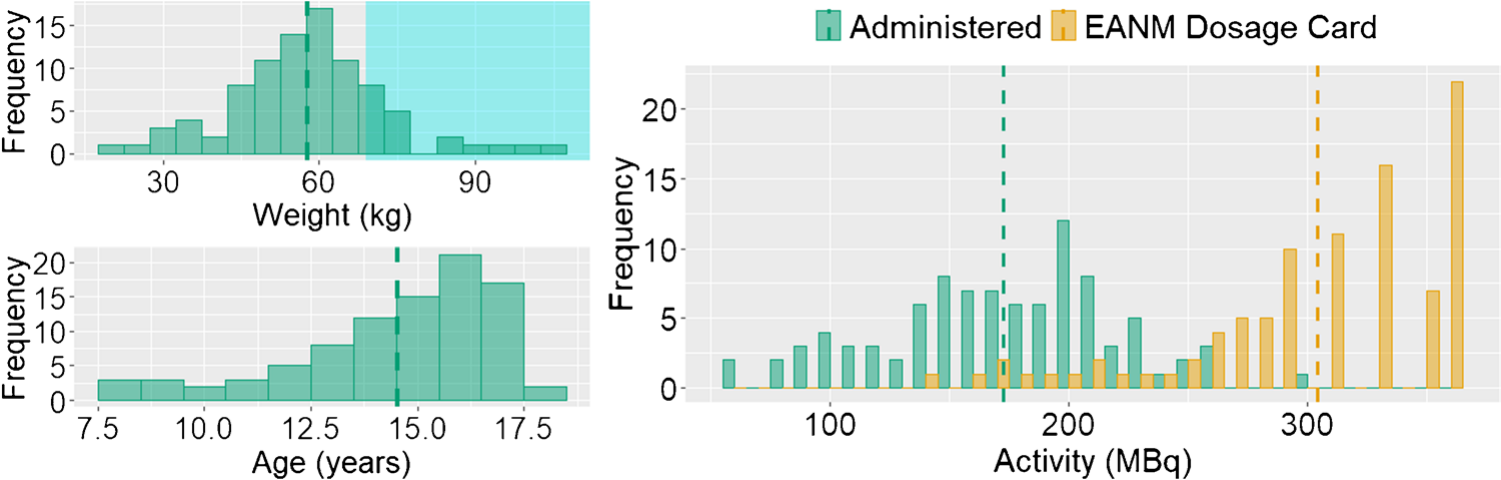
Baseline characteristics of the subset of 91 selected [^18^F]FDG scans from the EuroNet-PHL-C2 trial. Histograms of weight (top left, bin size = 5 kg), age (bottom left, bin size = 1 year) and administered activity as well as activity recommended by the EANM Paediatric Dosage Card (right, bin size = 10 MBq). Vertical dashed lines indicate the mean activities. The cyan box marks the weight range > 68 kg not defined by the EANM Paediatric Dosage Card

The characteristics of the subset (*n* = 91) are depicted in Fig. 1. The activity recommended by the EANM Paediatric Dosage Card had been reduced by a median of 45% (range 14–68%, Supplemental Table 2).

#### Homogeneity of the dataset

PET/CT data from four different vendors (CPS, General Electric, Philips and Siemens) were included in the analysis. While attenuation, scatter and decay correction had been applied to all data, other features such as time-of-flight reconstruction or post-reconstruction filtering were only partially used. An overview of the PET/CT imaging parameters potentially affecting image quality can be found in Supplemental Table 3.

#### Assessment of the image quality based on visual assessment (QSV)

**Fig. 2:**
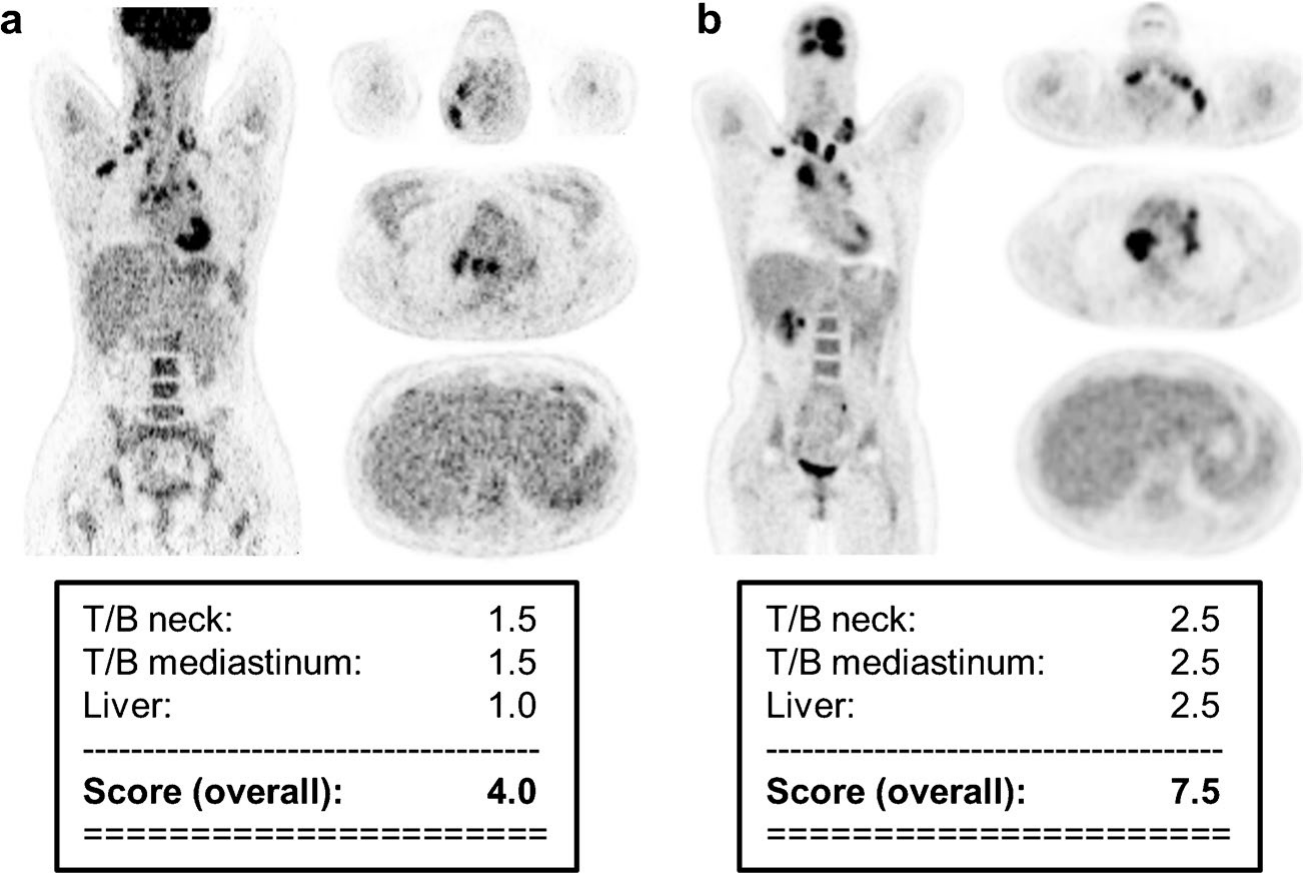
Examples of the visual assessment of image quality for still acceptable image quality (**a** QSV of 4.0) and very good image quality (**b** QSV of 7.5). A transversal slice (top left) and axial slices of the three areas considered in the assessment are shown: neck (top right), mediastinum (centre right) and liver (bottom right). The scores of the three areas considered as well as the total score are given at the bottom

All QSV values were ≥ 3 and thus found to be suitable for sufficient reporting. A histogram of the assigned QSV values divided among the four manufacturers is depicted in Supplemental Fig. 3. No statistically significant differences in QSV were found between Siemens and GE systems (*t*(37) = 0.85, *p* = 0.40), nor between Siemens and Philips systems (*t*(32) = 1.24, *p* = 0.22). As only six scans performed on CPS systems were available, these data were not included in the statistical analysis. To give a visual impression of the image quality, Fig. 2 shows two examples for a QSV of 4.0 and 7.5, respectively.

#### Noise analysis based on the coefficient of variation (CoV)

A noise analysis was performed based on the CoV in the 30 ml VOI (mean volume 30.2 ± 1.1 ml) of the healthy liver. Due to the wide range of influencing factors such as frame duration, voxel volume and activity-per-weight in relation to the size of the subset, however, the noise could not be adequately analysed by a univariate analysis. Instead, the visual quality assessment by two independent readers must suffice at this point, clearly indicating that all scans were appropriate for clinical evaluation. Further details on the influence of the individual factors can be found in Supplemental Fig. 4.

#### Investigation of the dependence of the tumour-to-background ratio (TBR) on the administered activity

**Fig. 3:**
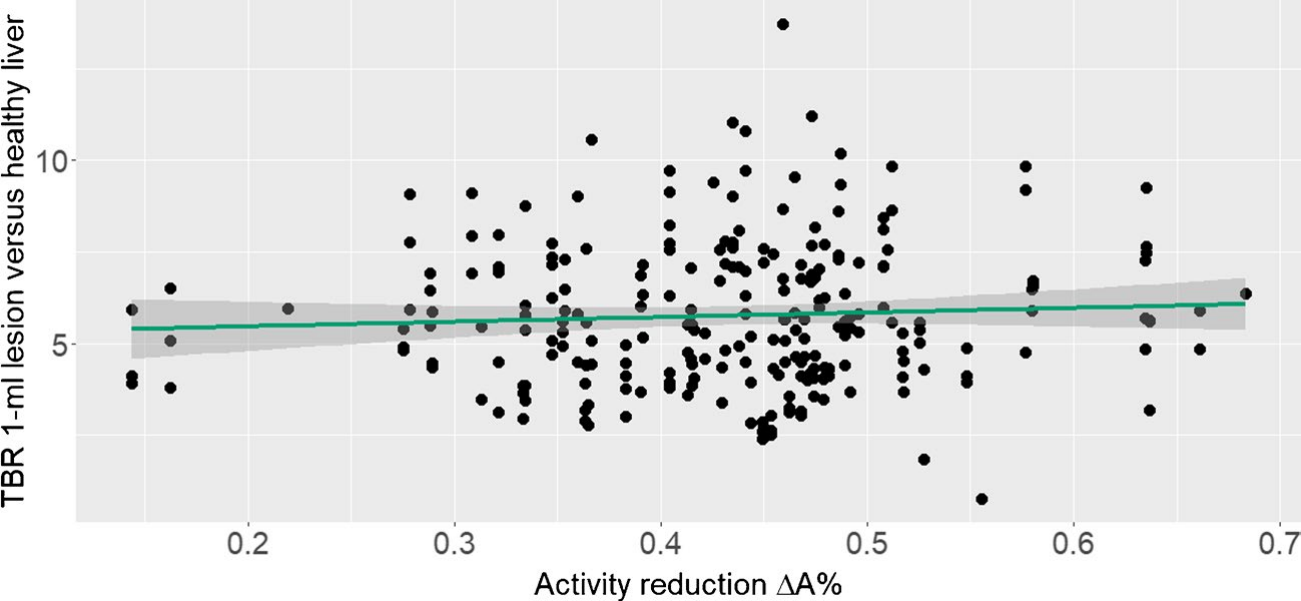
Linear regression analysis of the TBR in a 1-ml lesion (healthy liver background) based on the activity reduction $\Delta A\mathrm{\%}$ showing a very weak correlation (Pearson correlation coefficient, *r*(242) = 0.06, *p* = 0.352). The regression line (slope, 1.16 ± 1.35 MBq/kg and intercept, 5.21 ± 0.60 MBq) is plotted in green with 95% confidence intervals plotted in grey

A total of 244 lesions were analysed, resulting in a median TBR of 5.6 (range 0.8–13.7). Although the hypothesis of correlation could not be completely rejected (*p* = 0.352), the correlation was found to be very weak (Fig. 3, *r* = 0.06), inferring an almost negligible advantage of higher activities.

### Proposed correction for the EANM Paediatric Dosage Card

An upper limit of 3.7 MBq/kg, as given in the North American consensus guideline of the SNMMI [3], covers most of the data in the subset. A linear regression analysis between body weight and administered activity (slope, 3.24 ± 0.29 MBq/kg, intercept, − 9.16 ± 15.68 MBq, Supplemental Fig. 5) arrives at a similar conservative upper limit of 3.53 MBq/kg (slope plus one standard deviation). Consequently, this value represents an adequate basis for an update of the EANM Paediatric Dosage Card [1, 4]. To be consistent with the previous versions of the EANM Paediatric Dosage Card and the methodology described in [1, 2, 4, 6, 10], the reduced values for conventional [^18^F]FDG-PET/CT given in Table 1 are proposed for a frame duration of 3 min per bed position.

**Table 1:** Suggested update of the EANM Paediatric Dosage Card

Class	Baseline activity (MBq)	Minimum recommended activity (MBq)
B	15.8	16

A mean ratio of 0.61 ± 0.07 was found between a 3.7 MBq/kg activity regimen and the activities recommended by the existing EANM Paediatric Dosage Card. Consequently, the baseline value of 25.9 MBq provided by the dosage card for [^18^F]FDG torso was reduced by this factor. Table 2 shows the recommended activities and effective doses, taken from ICRP128 [11] for [^18^F]FDG, in comparison to an activity of 3.7 MBq/kg. In order to be consistent with the previous values, and due to the lack of data for effective doses according to the weighting factors of ICRP 103 [12], the tissue weighting factors of ICRP 60 were applied [13].

**Table 2:** Suggested update of the EANM Paediatric Dosage Card. Recommended administered activities (dosage card vs. weight-based) and effective dose values taken from ICRP 128 [11], for an acquisition with 3 min per bed position. For comparison, the values of the EANM Paediatric Dosage Card [4] and the North American consensus guideline are provided [3]

Age	Newborn	1 year	5 years	10 years	15 years	Adult
Weight (kg)	3	10	19	32	57	68
Activity (MBq)	16	43	80	115	184	221
Effective dose (mSv)	NA	4.1	4.3	4.3	4.4	4.2
For comparison:						
EANM Paediatric Dosage Card 3D mode [2] / [^18^F]FDG-PET of the brain [4]* (MBq)	14	38	65	102	163	200
EANM Paediatric Dosage Card 2D mode [2] / [^18^F]FDG of the torso [4]* (MBq)	26	70	120	189	302	363
North American consensus guideline lower limit of 3.7 MBq/kg [3] (MBq)	11	37	70	118	204	252
North American consensus guideline upper limit of 5.2 MBq/kg [3] (MBq)	26	52	98	166	295	352

^*^Interpolated values

This suggested update of the EANM Paediatric Dosage Card, based on the analysis presented in this work, results in a mean activity reduction by 39%.

**Fig. 4:**
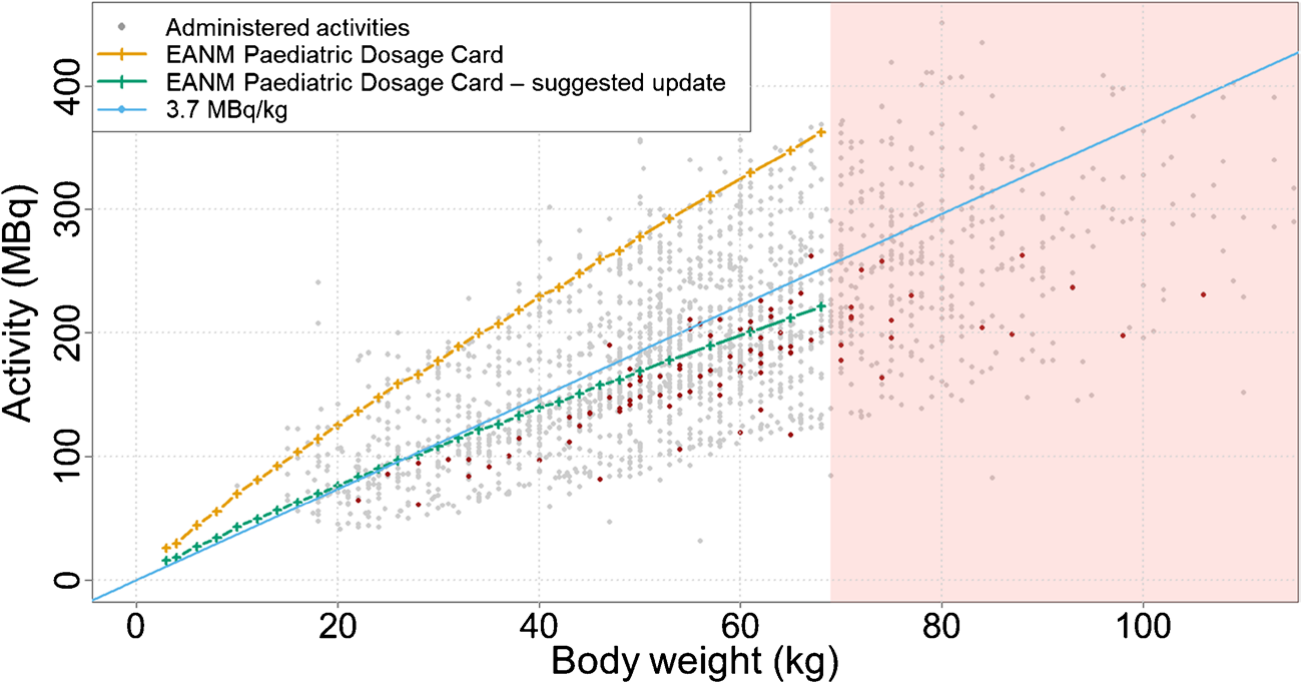
Activities recommended by the current EANM Paediatric Dosage Card (orange) [4] suggested update based on this work (green), and 3.7 MBq/kg activity regimen (blue line) as a function of body weight. In addition, the administered activities from all 2082 patient scans (grey dots) and the subset used in this study are given (dark red dots). Lastly, patients with body weight > 68 kg are indicated by the red box

Figure 4 shows the proposed activities to be administered based on the current EANM Paediatric Dosage Card [4], the recommended update developed in this work and the corresponding values of a 3.7 MBq/kg regimen as a function of body weight. To put these results in the context of the overall study, data from all 2082 PET/CT examinations are plotted.

The activity values proposed by this work are consistent with the recommendations by Dickson et al. [10] for PET/MR systems with higher sensitivity.

## Discussion

The present analysis comprises a subset of 2082 PET/CT scans from the EuroNet-PHL-C2 trial, a large multicentric clinical trial in a paediatric population. All 2082 PET/CT scans, 1960 of which were below the activity recommended by the EANM Paediatric Dosage Card by a median of 99.4 MBq, were evaluated by an independent evaluation board; none of the scans was classified as not eligible, suggesting good adherence to imaging guidelines [14] and showing that the activity recommendations of the latest version of the EANM Paediatric Dosage Card [4] can be reduced. To ensure the highest possible relevance of the present retrospective analysis, a subset of 91 patients was selected according to predefined criteria based on the highest absolute activity reduction, which should correspond to the poorest image quality.

For this analysis, we decided to include patients with an activity reduction based on absolute values instead of a relative reduction of the administered activity. When applying this criterion, only a very small part of the lightweight patients are included. However, this choice is justified as, with increasing weight, more attenuation and scatter are observed in the patients because of an increase of the soft tissue proportion (above all muscle mass/fat), with the consequence that an impairment of the image quality is to be expected. In addition, there is a larger distribution volume in this patient group, leading to a lower concentration of the radiopharmaceutical per body section in heavier patients. Both factors might lead to a much stronger decrease of the image quality in heavier patients compared to younger patients. Consequently, we chose to select PET/CT scans based on the highest absolute activity reduction, which should correspond to the poorest image quality.

Retrospective PET/CT data were accessible in DICOM format. Because the amount of acquisition and reconstruction information available in the respective DICOM headers was very different for the manufacturers considered, there was a wide variation in the number of parameters available. Consequently, some of the datasets could not be clearly assigned certain acquisition and reconstruction parameters, which would have been imperative for assessing the image quality in relation to the measurement parameters. As an example, only 45% of the datasets contained information on iterations, subsets and postfilter applied during reconstruction – parameters with major influence on the noise characteristics in the image. While the patient weight, which is needed for calculation of the standardised uptake value, SUV, had been entered for all patients, the patient height was only available for 59% of the patients, making it difficult to include the patients’ physique in the analysis. Due to these limitations in combination with the limited data size of *n* = 91, a detailed univariate analysis of individual parameters was not feasible, as each of the subgroups becomes too small to deliver statistically meaningful results. On a positive note, however, the data cover a wide range of possible clinical situations, making them ideal for deriving universally applicable recommendations. Therefore, all available data were included in our weight-based activity analysis.

The consistently good quality of the datasets, which can be seen in Fig. 4, allows two important observations: First, it shows that the activities proposed by the present version of the EANM Paediatric Dosage Card [4], and even the significantly reduced administered activities, exceed the amount of activity required for acceptable image quality. Second, it shows an unavoidable limitation of this study. To reliably explore the lower limit of data quality, a considerable proportion of the patients would have to be administered with lower activities (resulting in a lower image quality), which would, however, entail the risk of clinically unevaluable data, and would thus be ethically unacceptable. Although the data used in this work allow us to reduce the recommendation for the activity to be administered according to the EANM Paediatric Dosage Card, we do not necessarily reach an absolute lower limit.

In the last years, two publications studied, in single-centre settings with modern time-of-flight PET/CT systems (Siemens mCT), how the activity to be administered can be reduced in comparison to the EANM Paediatric Dosage Card [15, 16]. While both studies propose higher activity reductions than what is proposed in this work, one should note that both studies were based on state-of-the-art PET/CT equipment in combination with optimised acquisition and reconstruction settings. In contrast, our analysis is based on PET/CT images from a wide range of PET/CT systems from different manufacturers with different acquisition and reconstruction settings, resulting in more conservative, but also more universally applicable reduction recommendations.

It is important to note that these dosage recommendations should be taken in context of “good practice” for nuclear medicine and do not substitute for national and international legal or regulatory provisions. In addition, these recommendations represent a conservative limit needed to obtain a baseline quality necessary for diagnostic analysis of the image data. Lower activities might be conceivable if the PET/CT system including site-specific imaging and reconstruction parameters affecting image quality (e.g. frame duration or reconstruction parameters) suit the needs of the clinic with respect to their specific equipment, clinical preference and the particular needs of their patients.

## Conclusion

Based on our results, we suggest amended values for activities to be administered based on the EANM Paediatric Dosage Card for [^18^F]FDG-PET/CT scans with conventional PET/CT systems.

### Supplementary Information

Below is the link to the electronic supplementary material.

Supplementary file1 (PDF 850 KB)
