# Supplementary material for: Analysis of image data from the EuroNet PHL-C2 trial indicates a potential reduction in injected F-18 FDG activities in children: a proposal to update the EANM Paediatric Dosage Card
Source: Eur J Nucl Med Mol Imaging. 2023 Sep 20;51(2):405–11. doi: 10.1007/s00259-023-06396-w (PMC10774179; doi:10.1007/s00259-023-06396-w)
Supplement: Supplementary file 1 — Supplementary file1 (PDF 850 KB) [file 259_2023_6396_MOESM1_ESM.pdf]

Analysis of image data from the EuroNet PHL-C2 trial indicates a potential reduction in injected F-18 FDG activities in children: A proposal to update the EANM Paediatric Dosage Card – Supplemental Material

Authors:

Johannes Tran-Gia*[1], Uta Eberlein[1], Michael Lassmann[1], Christine Mauz-Körholz[2], Dieter Körholz[2], Pietro Zuccetta[3], Zvi Bar-Sever[4], Ute Rosner[5], Thomas Walter Georgi[5], Osama Sabri[5], Regine Kluge[5], Arnoldo Piccardo[6]**, Lars Kurch[5]**

* Corresponding author
** These authors contributed equally

***Corresponding Author**

Dr. rer. nat. Johannes Tran-Gia

Department of Nuclear Medicine

University Hospital Würzburg

Oberdürrbacher Str. 6

97080 Würzburg, Germany

Phone: +49-931-201-35421

Email: Tran_J@ukw.de

INTRODUCTORY REMARKS TO THIS SUPPLEMENT

This supplement provides background information on the individual analysis steps, such as definitions, figures. In addition, it contains more detailed results of the statistical evaluations.

METHODS

The EuroNet-PHL-C2 trial – General information

Within the EuroNet-PHL-C2 trial each patient received [^{18}F]FDG-PET for initial staging and for early response assessment following two courses of an intensive chemotherapy. Patients in intermediate and advanced stages who did not show an adequate early metabolic response received another [^{18}F]FDG-PET scan after completion of chemotherapy [9].

Nearly all [^{18}F]FDG-PET images were centrally reviewed at Leipzig University Hospital. For this purpose, the original image data were transferred and stored onto a central server [8].

The EuroNet-PHL-C2 study protocol did not define mandatory criteria of how to perform the [^{18}F]FDG-PET scans. Given the rarity of the disease (not more than 10 patients per centre and year) and the large number of recruiting centres, strict regulations would not have been practicable, either. Thus, the image data closely reflect the clinical reality in various PET centres. In order to provide the PET centres with some key information, a separate imaging manual was available which included recommendations on how to prepare the patient and how to perform the [^{18}F]FDG scan. Concerning the individual [^{18}F]FDG activity to be administered, it was recommended to consult the EANM Paediatric Dosage Card.

Data included in the analysis

For each of the 91 [^{18}F]FDG-PET scans from the subset, the following additional data were recorded in a separate Excel sheet:

- Sex, age and weight of the patients;
- PET centre at which the scan was performed;

- Administered activity ($A_{\text{Administered}}$) in MBq;
- Recommended activity ($A_{\text{EANM Paediatric Dosage Card}}$) in MBq from the EANM Paediatric Dosage Card;
- Activity reduction (ΔA):

$$\Delta A = A_{\text{EANM Paediatric Dosage Card}} - A_{\text{Administered}} \;;$$

- Activity reduction (ΔA%) in percent:

$$\Delta A\% = \frac{\Delta A}{A_{\text{EANM Paediatric Dosage Card}}} \cdot 100 \;;$$

- Vendor of the PET scanner;
- Time interval from tracer application until the start of image acquisition;
- Information on attenuation, scatter and decay correction;
- Information on applied postfilter if specified in the header;
- Voxel volume in mL;
- Time per bed position in minutes (min);
- Number of iterations and subsets if specified in the header;

All metadata were either extracted from the DICOM headers of the image data or taken from the database of the EuroNet-PHL-C2 study.

Tumour-to-background ratio (TBR) calculation

The tumour-to-background ratio (TBR) was estimated based on the ratio between lesions and the background in the healthy liver tissue. For this purpose, a dedicated image data analysis was performed. More specifically, mean of the standardized uptake value (SUV) in sphere volumes of interest (VOIs) of 1 ml volume around up to 3 lesions (minimum of 1) per patient was used as “tumour”. Similarly, the mean SUV in sphere VOIs of 30 ml volume in the healthy liver tissue was used as “background”.

The tumour-to-background ratio (TBR) between each lesion and the liver background for the corresponding patient was then calculated as:

$$TBR = \frac{Mean\ SUV\ (1\text{-}ml\ lesion\ VOI)}{Mean\ SUV\ (30\text{-}ml\ liver\ VOI)} \;;$$

Coefficient of variation (CoV) calculation

The mean and standard deviation of the SUV in a sphere VOI of 30 ml volume in the healthy liver tissue were used to calculate the coefficient of variation (CoV) as the ratio between standard deviation in SUV by the mean SUV inside the VOI:

$$CoV = \frac{Standard\ deviation\ SUV\ (30\text{-}ml\ liver\ VOI)}{Mean\ SUV\ (30\text{-}ml\ liver\ VOI)};$$

Visual quality score (QSV) calculation

As a quantitative measure for the visual assessment of image quality, a visual quality score (QSV) was defined as follows: First, a score was assigned to each item according to Supplemental Table 1. The sum of the scores for items 1 to 3 resulted in the QSV (value range between 0 and 9). A total score between 7.0 and 9.0 indicates excellent image quality, between 4.0 and 6.5 good image quality, and between 2.0 and 3.5 reduced image quality, respectively. In the latter case, the PET scan is still usable for clinical reporting with caution. In contrast, a total score between 0 and 1.5 indicates inadequate image quality, which is not suitable for clinical reporting.

Supplemental Table 1: Details on the image quality score established based on a visual assessment of [^{18}F]FDG-PET scans (quality score visual, QSV)

**Items and description**	**Score**
1 Homogeneity of the liver uptake	
Smooth, not noisy	3.0
Slightly noisy	2.0
Moderately noisy	1.0
Strongly noisy	0
2 Impression tumour / background (neck)	
Very high contrast	3.0
High contrast	2.0
Moderate contrast	1.0
Low contrast	0.5
Very low – no contrast	0
3 Impression tumour / background (mediastinum)	
Very high contrast	3.0
High contrast	2.0
Moderate contrast	1.0
Low contrast	0.5
Very low – no contrast	0

RESULTS

Characteristics of the 2,082 consecutive [^{18}F]FDG-PET scans

The baseline characteristics of the 2,082 [^{18}F]FDG scans performed are illustrated in Supplemental Figure1.

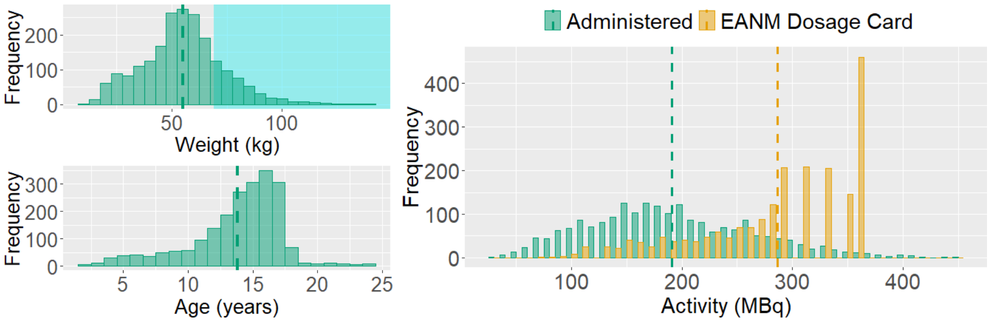

Supplemental Figure 1: Baseline characteristics of the 2,082 [^{18}F]FDG scans performed within the EuroNet-PHL-C2 trial. Histograms of weight (top left, bin size = 5 kg), age (bottom left, bin size = 1 year), and administered activity as well as activity recommended by the EANM Paediatric Dosage Card (right, bin size = 10 MBq). Vertical dashed lines indicate the mean activities. The cyan box marks the weight range > 68 kg not defined by the EANM Paediatric Dosage Card.

The fact that 48 of the 2,082 patients (2.3%) were between 18 and 24 years old can be explained by the fact that in some countries, such as Italy and the UK, patients up to an age of 24 were recruited for the study. This is due to the fact that the cut-off to distinguish between adolescents and young adults at the age of 18 years is arbitrary, as the histology and the biologic behaviour of Hodgkin lymphoma do not differ in these two populations.

Analysis of the selected subset of 91 [^{18}F]FDG-PET scans

a) Characteristics of the subset

In addition to Figure 1 of the main manuscript, the specific characteristics of the subset (n=91) are summarised in Supplemental Table 2. In addition, a linear regression analysis between activity reduction $\Delta A\%$ and patient weight revealed a strong negative correlation (Pearson correlation coefficient r(89)=−.37, $p<10^{-3}$, Supplemental Figure 2). Since patient weight and age are also strongly correlated (r(89) = .68, $p<10^{-13}$), this implies that the activity for younger patients had been reduced to a greater extent.

Supplemental Table 2: Baseline characteristics of the subset

**Patient characteristics**	**N**
Number of [^{18}F]FDG-PET scans (equal to patients)	91
Centres involved	43 (1-5 scans per PET centre)
Sex<br>Female<br>Male	 46 (49%)<br>45 (51%)
Median age, years	15 (8-18)
Median weight, kg	58 (22-106)
Median $A_{\text{Administered}}$, MBq	173 (61-263)
Median $A_{\text{EANM Paediatric Dosage Card}}$, MBq	311 (137-363)
Median ΔA, MBq	128 (72-230)
Median $\Delta A\%$, percent	45% (28-68%)

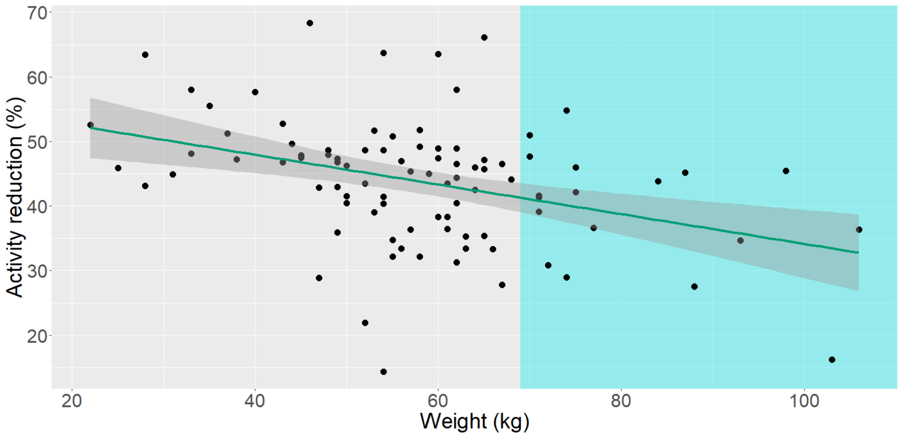

Supplemental Figure 2: Linear regression analysis of the activity reduction $\Delta A\%$ based on the patient weight (kg) for the subset. Fitted regression line: $\Delta A\%$ (weight) = − 0.23% * weight / kg + 57.16%. Pearson correlation coefficient of r(89) = −.37, p < 10^{-3}. The cyan box marks the weight range > 68 kg not defined by the EANM Paediatric Dosage Card, which was nonetheless included in the analysis. The regression line is plotted in green with 95% confidence intervals plotted in grey.

b) Homogeneity of the dataset

To visualise the heterogeneity of the imaging parameters, Supplemental Table 3 lists the imaging parameters potentially affecting image quality.

Supplemental Table 3: Homogeneity of the PET/CT imaging parameters

PET/CT imaging characteristics	N
PET/CT scans included per vendor<br>CPS<br>General Electric<br>Philips<br>Siemens	 6<br>23<br>17<br>45
Mean time interval between injection and imaging, minutes	66±14 (42-131)
Median voxel volume, mm^3	64 (9-98)
Median time per bed position, minutes	2.5 (0.8-4.0)
Number of iterations	3 (2-4)
Number of subsets	12 (8-24)
Postfilter applied<br>None<br>2mm Gaussian<br>4mm Gaussian<br>5mm Gaussian<br>Not indicated	 3 (3%)<br>18 (20%)<br>4 (4%)<br>25 (27%)<br>41 (45%)
Time of flight available?	34 (37%)

c) Assessment of the image quality based on visual assessment (QSV)

In addition to the explanations given in the main document, Supplemental Figure 3 shows a histogram of the assigned QSV values divided among the four manufacturers.

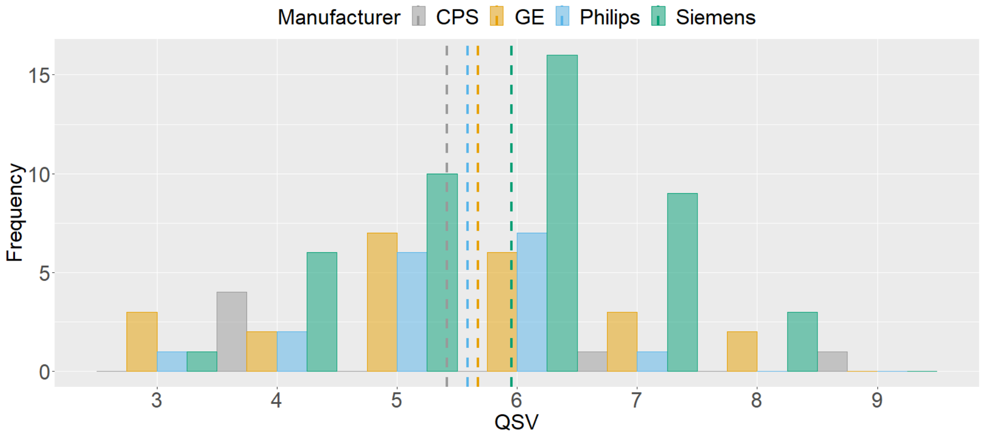

Supplemental Figure 3: Histogram of assigned QSV values divided into the PET/CT manufacturers used for the acquisition (bin size = 1). The mean QSV values for each manufacturer are indicated by the vertical dashed lines. Note that each bin contains four colours: grey, yellow, blue and green from left to right.

d) Noise analysis based on the CoV

The scatter plots with linear regression lines in Supplemental Figure 4 visualize the dependency of the noise on frame duration (acquisition duration per bed position), voxel volume, weight, and administered activity per body weight.

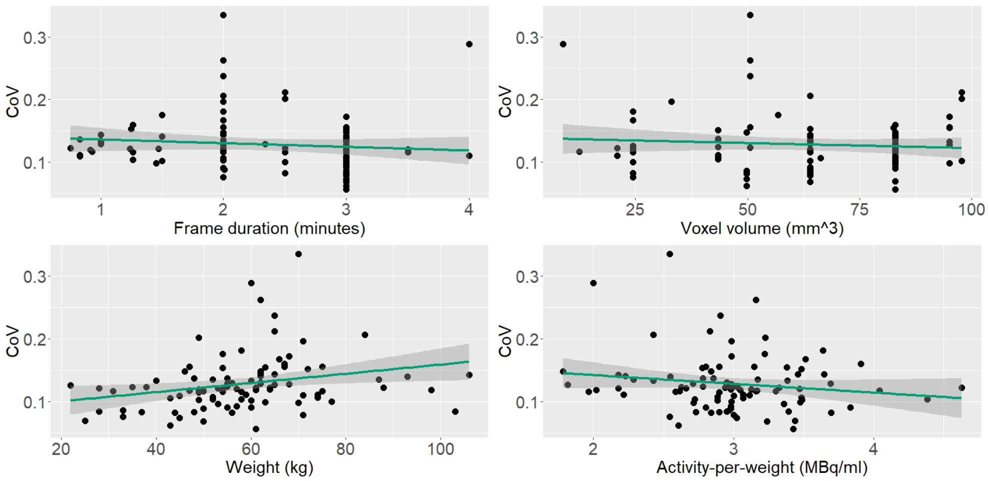

Supplemental Figure 4: Scatter plots of the CoV versus frame duration (top left), voxel volume (top right), weight (bottom left), and administered activity per body weight (bottom right). Regression lines are plotted in green with 95% confidence intervals plotted in grey.

Although weak negative correlations were found between CoV and frame duration (r(89) = -.10), voxel volume (r(89) = -0.08), and activity-per-weight (r(89) = -.16), it was only statistically significant for the body weight based on a Pearson correlation test (r(89) = .25, p < .02). Especially for the discrete parameters frame duration and voxel volume, a large range of CoV values occurred for individual values (e. g., a frame duration of 2 minutes with median 0.13 and range 0.08-0.33, or a voxel volume of 83 mm^3 with median 0.12 and range 0.06-0.16). This indicates that the noise cannot be adequately analysed by a univariate analysis.

Proposed update for the EANM Paediatric Dosage Card

The linear regression analysis between body weight and administered activity is given in Supplemental Figure 5. With a slope of 3.24±0.29 MBq/kg, it arrives at a conservative upper limit of 3.53 MBq/kg (slope plus one standard deviation), supporting our proposal of a new upper limit of 3.7 MBq/kg.

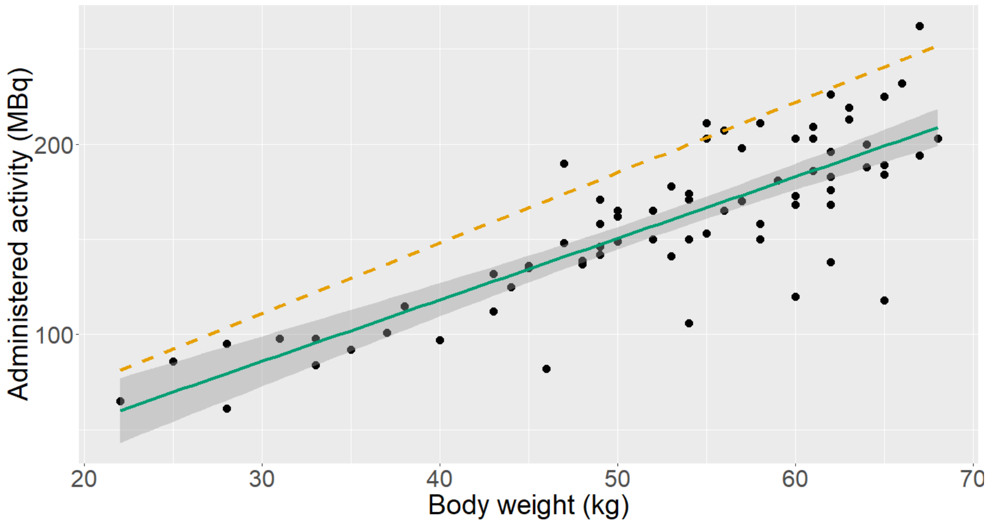

Supplemental Figure 5: Regression analysis between body weight and administered activity (slope, 3.24±0.29 MBq/kg; intercept, -9.16±15.68 MBq). The area shaded in grey corresponds to the 95% confidence interval. The orange line corresponds to a weight-dependent activity of 3.7 MBq/kg.
